# Conventional screening measure does not accurately capture screening status in a minority of patients with colorectal cancer

**DOI:** 10.1097/MD.0000000000043593

**Published:** 2025-07-18

**Authors:** Taylor M. McCready, Shinjini Nandi, Yingzhi Qian, Shawn Wen, Simona C. Kwon, Ann G. Zauber, Jason A. Dominitz, Scott E. Sherman, Peter S. Liang

**Affiliations:** a Department of Population Health, NYU Grossman School of Medicine, New York, NY; b Department of Mathematical Sciences, Montana State University, Bozeman, MT; c Department of Medicine, NYU Grossman School of Medicine, New York, NY; d Department of Epidemiology & Biostatistics, Memorial Sloan Kettering Cancer Center, New York, NY; e National Gastroenterology and Hepatology Program, Veterans Health Administration, Washington, DC; f Department of Medicine, University of Washington School of Medicine, Seattle, WA; g Department of Medicine, VA New York Harbor Health Care System, New York, NY.

**Keywords:** colorectal cancer, screening, Veteran

## Abstract

Colorectal cancer (CRC) screening uptake in the Veterans Health Administration (VA) has been reported to be higher than the US general population, but CRC remains a prevalent cancer within the VA system. To examine CRC predictors and the extent to which the conventional definition of up-to-date screening applies to the population, we conducted a case-control study using VA data from 2012 to 2018. We classified patients into 5 categories: up-to-date or not up-to-date average-risk patients aged 50 to 75 (Categories 1 and 2), up-to-date or not up-to-date average-risk patients aged <50 or >75 (Categories 3 and 4), and high-risk patients (Category 5). Each CRC case was matched by age, sex, and facility with 4 controls. We performed multivariable conditional logistic regression, adjusting for race and ethnicity, diabetes, obesity, and alcohol use. Among 3714 CRC cases identified, Category 4 (odds ratio [OR] 1.40, 95% CI 1.11–1.78) and Category 5 (OR 6.23, 95% CI 5.06–7.66) patients had a higher risk of CRC compared to Category 1 patients. Compared with White patients, Black patients had a higher risk (OR 1.54, 95% CI 1.37–1.73). Diabetes (OR 1.65, 95% CI 1.51–1.81) and alcohol use disorder (OR 1.53, 95% CI 1.35–1.73) were also associated with CRC. Most CRC cases occurred in individuals aged 50 to 75, but 12.5% occurred in persons who were outside of this age range or had high-risk personal or family history. The conventional measure of CRC screening, focused on average-risk individuals aged 50 to 75, does not reflect screening status in an important minority of CRC patients.

## 1. Introduction

Colorectal cancer (CRC) is a significant health concern in the US, accounting for 7.6% of all new cancer cases.^[1]^ In 2025, an estimated 154,270 people will be diagnosed with CRC, and 52,900 will die, making it the third most common cancer in both men and women and the second leading cause of cancer death.^[1]^ The consensus approach to CRC prevention is to increase screening uptake, which is currently 72% among individuals aged 50 to 75 years in national surveys.^[2–4]^ This stands in contrast to the screening rate among patients receiving care from the Veterans Health Administration (VA), which is substantially higher. The published uptake in the VA was 72% in 2004 and has exceeded 80% since 2009.^[5]^ Despite this, CRC persists as the third most common and deadliest cancer in the VA.^[6]^

Screening is estimated to account for half of the reduction in CRC incidence and mortality observed in the US.^[4,7]^ However, a cross-sectional study found that the stage at diagnosis and survival for Veterans were similar to that of the comparatively under-screened general population.^[6]^ There are several possible explanations for why some individuals may still develop CRC despite following recommended screening or surveillance guidelines, including a high proportion of disease occurring in individuals who are outside the recommended age range for screening,^[8]^ disease limited to the unscreened population, failure of the screening test, or a large number of individuals whose adherence to screening and surveillance guidelines is not captured by the conventional screening metric. The essential question is whether the current strategy and metrics for preventing CRC, both in the VA and elsewhere, are optimal.

Understanding which patients are diagnosed with CRC despite being in a healthcare system with high screening rates is crucial to evaluate the effectiveness of the current CRC prevention metric. In this study, we sought to categorize Veterans diagnosed with CRC based on whether they were captured by the conventional screening definition, and the reason why, if not. We examined predictors of CRC diagnosis and whether the conventional measure of screening, in average-risk individuals aged 50 to 75, adequately captures screening status in all patients with CRC.

## 2. Methods

### 2.1. Study design

This study was approved by the VA New York Harbor Health Care System Institutional Review Board (study 1709), New York, on January 7, 2019, and the NYU Langone Health Institutional Review Board (study 19-00023), New York, on January 10, 2019.

We performed a case-control study using VA data from 2012 to 2018. To identify cancer cases and controls, we used the Corporate Data Warehouse, which is a repository for all clinical and structured data from the electronic medical record.

We identified all cases of CRC from 2 VA medical centers (VA New York Harbor Health Care System and VA Puget Sound Health Care System) between 2012 and 2018. For each individual diagnosed with CRC (case), we identified all individuals without CRC who matched the case on 3 variables – age (±3 yr), sex, and facility site – and randomly selected 4 matched controls. Each control was used once.

### 2.2. Screening status and predictors

Patients were classified into 5 categories based on their screening status: average-risk patients aged 50 to 75 years who were up to date (Category 1) or not up to date (Category 2) with screening, average-risk patients who were outside the recommended screening age range during the study period (age <50 or >75 yr) who were up to date (Category 3) or not up to date (Category 4) with screening, and high-risk patients with inflammatory bowel disease, hereditary cancer syndromes, or family history of CRC (Category 5). While guidelines have since changed to lower the starting screening age from 50 to 45 years,^[9]^ the 50 to 75 year age range used in this analysis reflected the national guidelines during the study period. Patients were considered to be up to date if they received any test recommended by the US Preventive Services Task Force within the appropriate time frame,^[9]^ including colonoscopy within the last 10 years and guaiac-based fecal occult blood test or fecal immunochemical test within the last year. Individuals who were outside the recommended screening age range were also considered up to date if they had completed a screening test within the corresponding time frame. Patients were considered average-risk if they did not have documented inflammatory bowel disease, hereditary cancer syndromes, or a family history of CRC based on the following International Classification of Diseases (ICD) codes: ICD-9 555.x-556.x, 759.6, V84.09; ICD-10 K50.x-K51.x, Q85.8, Z15.09.

We evaluated 4 additional predictors to measure the association between screening status and CRC diagnosis: race and ethnicity, diabetes, obesity, and alcohol use disorder. The race and ethnicity categories were non-Hispanic White (White), non-Hispanic Asian (Asian), non-Hispanic Black or African American (Black), Hispanic or Latino, and Other or not reported. Obesity was defined based on ICD code or a measured body mass index of 30 or greater. Diabetes and alcohol use disorder were ascertained by ICD codes and coded as binary variables.

### 2.3. Statistical analysis

To evaluate the link between screening category and CRC, we developed multivariable conditional logistic regression models that adjusted for race and ethnicity, diabetes, obesity, and alcohol use disorder. We compared demographic characteristics between case and control patients using the chi-squared test. A sensitivity analysis restricted to individuals who had a primary care provider (PCP) appointment within 2 years of the case’s date of diagnosis was also conducted. Results were considered statistically significant if the 2-sided *P* value was <.05. All data were analyzed using R (R Foundation for Statistical Computing, Vienna, Austria).

## 3. Results

A total of 18,570 people were included in the analysis, with 3714 CRC cases and 14,856 controls (Table 1). The study population was predominantly male (97.1%), and the average age was 69 years. Among cases, there were 609 individuals (16.4%) in Category 1, 2641 individuals (71.1%) in Category 2, and 290 individuals (7.8%) in Category 5. The majority of CRC patients were White (55.0%), followed by Other/Not Reported (23.9%) and Black (15.8%). For comorbidities, the prevalence of diabetes (26.7% vs 17.9%), obesity (32.4% vs 29.4%), and alcohol use disorder (13.5% vs 9.3%) was higher among cases than controls (*P* < .001 for all).

**Table 1:** Patient characteristics.

Characteristic	Cases	Controls	*P* value	Total
	N = 3714	N = 14,856		N = 18570
Male, n (%)	3607 (97.1)	14,428 (97.1)	1.00	18,035 (97.1)
Category*, n (%)			<.001	
1	609 (16.4)	2640 (17.8)		3249 (17.5)
2	2641 (71.1)	11,391 (76.7)		14,032 (75.6)
3	37 (1.0)	121 (0.8)		158 (0.9)
4	137 (3.7)	485 (3.3)		622 (3.3)
5	290 (7.8)	219 (1.5)		509 (2.7)
Race and ethnicity, n (%)			<.001	
Asian	28 (0.8)	161 (1.1)		189 (1.0)
Black or African American	587 (15.8)	1834 (12.3)		2421 (13.0)
Hispanic or Latino	166 (4.5)	719 (4.8)		885 (4.8)
Other or not reported	889 (23.9)	1996 (13.4)		2885 (15.5)
White	2044 (55.0)	10,146 (68.3)		12,190 (65.6)
Diabetes, n (%)	991 (26.7)	2654 (17.9)	<.001	3645 (19.6)
Obesity, n (%)	1204 (32.4)	4365 (29.4)	<.001	5569 (30.0)
Alcohol use disorder, n (%)	502 (13.5)	1384 (9.3)	<.001	1886 (10.2)

* Categories: (1) Age 50 to 75 and up-to-date with screening; (2) Age 50 to 75 and not up-to-date with screening; (3) Age < 50 or >75 and up-to-date with screening; (4) Age < 50 or >75 and not up-to-date with screening; (5) individuals with high-risk factors (inflammatory bowel disease, hereditary cancer syndrome, family history of colorectal cancer).

On multivariable logistic regression, both Category 4 (odds ratio [OR] 1.40, 95% CI 1.11–1.78) and Category 5 (OR 6.23, 95% CI 3.58–7.66) patients had an increased risk of CRC compared to Category 1 patients (Table 2). There was no statistically significant association observed between the other categories and CRC diagnosis. Compared to White patients, Black patients had a higher risk (OR 1.54, 95% CI 1.37–1.73) of CRC. Additionally, diabetes (OR 1.65, 95% CI 1.51–1.81) and alcohol use disorder (OR 1.53, 95% CI 1.35–1.73) were both associated with an increased risk of CRC. Obesity (OR 1.06, 95% CI 0.98–1.15) was not significantly associated with CRC.

**Table 2:** Association between patient characteristics and colorectal cancer among 3714 cases and 14,856 controls.

Characteristic	Adjusted odds ratio (95% CI)
Category*	
1	Reference
2	1.11 (0.99–1.24)
3	1.39 (0.92–2.09)
4	1.40 (1.11–1.78)
5	6.23 (5.06–7.66)
Race and ethnicity	
White	Reference
Asian	0.87 (0.58–1.32)
Black or African American	1.54 (1.37–1.73)
Hispanic or Latino	1.09 (0.90–1.31)
Other or not reported	2.22 (2.02–2.44)
Diabetes	1.65 (1.51–1.81)
Obesity	1.06 (0.98–1.15)
Alcohol use disorder	1.53 (1.35–1.73)

* Categories: (1) Age 50 to 75 and up-to-date with screening; (2) Age 50 to 75 and not up-to-date with screening; (3) Age < 50 or >75 and up-to-date with screening; (4) Age < 50 or >75 and not up-to-date with screening; (5) individuals with high-risk factors (inflammatory bowel disease, hereditary cancer syndrome, family history of colorectal cancer).

We also conducted a sensitivity analysis on 4525 patients (4 controls for each of 905 cases) who had a PCP appointment within 2 years of the case’s diagnosis (Table 3). The results of this analysis revealed that, unlike the primary analysis, only Category 5 patients had a significantly increased risk of CRC (OR 5.29, 95% CI 3.46–8.10). The association between Black individuals and CRC (OR 1.33, 95% CI 1.01–1.74) was slightly attenuated compared to the primary analysis. However, associations for diabetes (OR 5.63, 95% CI 4.47–7.10) and alcohol use disorder (OR 7.25, 95% CI 5.54–9.50) were strengthened, and the association for obesity (OR 1.98, 95% CI 1.66–2.35) became significant in the sensitivity analysis.

**Table 3:** Sensitivity analysis of 4525 patients with PCP appointments within 2 years of diagnosis.

Characteristic	Adjusted odds ratio (95% CI)
Category†	
1	Reference
2	1.15 (0.97–1.38)
3	*
4	0.70 (0.12–3.89)
5	5.29 (3.46–8.10)
Race and ethnicity	
White	Reference
Asian	1.02 (0.42–2.49)
Black or African American	1.33 (1.01–1.74)
Hispanic or Latino	1.13 (0.74–1.73)
Other or not reported	1.40 (1.08–1.83)
Diabetes	5.63 (4.47–7.10)
Obesity	1.98 (1.66–2.35)
Alcohol use disorder	7.25 (5.54–9.50)

PCP = primary care provider.

* Data suppressed due to nonconvergence.

† Categories: (1) Age 50 to 75 and up-to-date with screening; (2) Age 50 to 75 and not up-to-date with screening; (3) Age < 50 or >75 and up-to-date with screening; (4) Age < 50 or >75 and not up-to-date with screening; (5) individuals with high-risk factors (inflammatory bowel disease, hereditary cancer syndrome, family history of colorectal cancer).

## 4. Discussion

In this large case-control study evaluating the association between screening category and CRC, a large majority of CRC cases (71.1%) occurred in individuals who were aged 50 to 75 years and not up to date with screening. However, 12.5% of cases occurred in persons who were either outside of this age range or were at higher risk for CRC based on personal or family history. With respect to medical comorbidities, diabetes and alcohol use disorder were significant risk factors for CRC. Furthermore, the results indicated significant racial disparities, with Black individuals having a higher risk for CRC compared to White individuals. These results underscore the limitations of a one-size-fits-all approach to CRC screening and highlight the need for more tailored strategies that consider individual risk factors.

To our knowledge, this is the first study to classify patients who have been diagnosed with CRC into distinct categories based on age, screening status, and personal risk for CRC. This analysis provides a novel perspective on the current approach used to calculate CRC screening uptake and identifies a shortcoming in the method. Our findings also partially explain why CRC incidence remains high despite dramatic improvements in screening uptake. The majority of CRC cases belong to categories that are captured by the conventional definition of screening adherence, but an important minority (12.5%) of cases fall outside of this definition. This highlights the limitations of the definition for screening adherence, particularly as CRC incidence is increasing among those younger than 50 years.^[10]^

While individuals with high-risk personal or family history have distinct recommendations with respect to screening intervals,^[11]^ the conventional screening measure does not account for this difference. Current screening uptake in the 50 to 75 year age group is 72% based on national surveys,^[2,3]^ but these figures overestimate the proportion of individuals who are up to date with guideline-concordant intervals for screening or surveillance, because the high-risk population should undergo colonoscopy more frequently than every 10 years.^[11]^ Our findings highlight the importance of regular CRC screening for individuals aged 50 to 75 years, as well as the need for earlier and more frequent screening for individuals with high-risk personal or family history.

If we exclude high-risk individuals (Category 5) and assess average-risk individuals based on screening status regardless of age, then 18.9% of patients were diagnosed with CRC despite being up to date with screening (Categories 1 and 3). These results are analogous to findings from 2 Kaiser Permanente health systems with high screening uptake, in which 24% of patients who died of CRC were up to date with screening.^[12]^ Incident CRC in the screened population can be attributed to 2 reasons: test failure – when a screening test does not detect an existing cancer, or interval cancer – when a cancer develops de novo between screening tests. Although we did not have the data in this analysis to distinguish between these 2 possibilities, our findings demonstrate that the protective effect of screening has limits. While screening detects most CRCs, no test is 100% sensitive, and a small proportion of CRCs will be missed.

Our findings on diabetes and heavy alcohol use are consistent with the large body of literature that implicates these modifiable risk factors in CRC development.^[13]^ As the VA is not a closed system, Veterans may receive screening at non-VA medical facilities and present for VA care after being diagnosed with a serious illness. In this situation, there are fewer opportunities to document important covariates, such as race and ethnicity, and comorbidities. To help account for this possibility, we conducted a sensitivity analysis restricted to individuals who had a PCP visit in the previous 2 years. This showed that the association between Category 4 patients and CRC became non-significant, and the risk estimate for Black individuals was attenuated. On the other hand, the risk estimates for diabetes and alcohol use disorder both strengthened, and the association for obesity became statistically significant. We cannot exclude the possibility of residual confounding.

The strengths of this study include the novel categories for screening status and a large sample size from the largest integrated healthcare system in the US. We also acknowledge a couple of limitations. First, since individuals who receive care in the VA healthcare system are predominantly male, older, and have more comorbidities when compared to the US general population,^[14,15]^ our results may not be broadly generalizable. Future research should replicate this analysis in other populations. Second, per convention, we defined being up to date with screening cross-sectionally relative to a single point in time and did not assess longitudinal screening adherence. The consistency of screening over time, beyond the period captured by the cross-sectional definition of screening, is likely also important for defining CRC risk.

## 5. Conclusion

In a large case-control study, most CRC cases were diagnosed in individuals aged 50 to 75 years who were not up-to-date with screening, but 12.5% of cases were identified in individuals outside of this age range or who were at higher risk for CRC based on personal or family history. High-risk individuals had a greater risk of CRC when compared to those who were up-to-date with screening and aged 50 to 75 years. The conventional definition of screening adherence, which only considered average-risk individuals aged 50 to 75 years during the study period and does not account for the recommendation for more intensive screening and surveillance for higher-risk individuals, overestimates the proportion of the population that is adherent to screening. This provides important context for understanding why the burden of CRC remains high despite substantial improvements in screening uptake.

## Author contributions

**Conceptualization:** Shinjini Nandi, Yingzhi Qian, Scott E. Sherman, Peter S. Liang.

**Data curation:** Shinjini Nandi, Yingzhi Qian.

**Formal analysis:** Shinjini Nandi, Yingzhi Qian.

**Funding acquisition:** Peter S. Liang.

**Investigation:** Taylor M. McCready, Shinjini Nandi, Yingzhi Qian, Shawn Wen, Scott E. Sherman, Peter S. Liang.

**Methodology:** Shinjini Nandi, Peter S. Liang.

**Project administration:** Peter S. Liang.

**Supervision:** Peter S. Liang.

**Writing – original draft:** Taylor M. McCready, Shawn Wen, Peter S. Liang.

**Writing – review & editing:** Taylor M. McCready, Shinjini Nandi, Yingzhi Qian, Shawn Wen, Simona C. Kwon, Ann G. Zauber, Jason A. Dominitz, Scott E. Sherman, Peter S. Liang.
